# VE-cadherin RGD motifs are dispensable for cell–cell junctions, endothelial barrier function and monocyte extravasation

**DOI:** 10.1080/21688370.2025.2478349

**Published:** 2025-03-18

**Authors:** Rianne M. Schoon, Werner J. van der Meer, Anne-Marieke D. van Stalborch, Jaap D. van Buul, Stephan Huveneers

**Affiliations:** a Amsterdam UMC, University of Amsterdam, Amsterdam, The Netherlands; b Swammerdam Institute of Life Sciences, University of Amsterdam, Amsterdam, The Netherlands

**Keywords:** Adherens junctions, barrier integrity, integrins, leukocyte extravasation, RGD motif, VE-cadherin

## Abstract

VE-cadherin is a key transmembrane protein involved in endothelial cell–cell junctions, playing a crucial role in maintaining vascular integrity and regulating selective leukocyte extravasation into inflamed tissue. The extracellular domain of human VE-cadherin contains two arginine-glycine-aspartate (RGD) motifs, which are known integrin-binding sites within extracellular matrix proteins, particularly for integrins of the β1, β3, and β5 families. In this study, we examined the functional relevance of these RGD motifs by generating VE-cadherin variants in which the RGD sequences were mutated to nonfunctional RGE. Immunofluorescence analysis showed that the VE-cadherin [D238E], VE-cadherin [D301E], and double-mutant VE-cadherin [D238/301E] variants formed stable endothelial cell–cell junctions that were comparable to junctions based on wild-type VE-cadherin. Additionally, electric cell-substrate impedance sensing (ECIS) confirmed that endothelial cells expressing each VE-cadherin RGD>RGE variant maintained efficient barrier function capacity. Moreover, monocyte transmigration assays demonstrated that the RGD>RGE mutations did not affect monocyte-endothelial interactions during transmigration. In summary, our findings indicate that the VE-cadherin RGD motifs are not essential for endothelial junction formation or monocyte transmigration.

## Introduction

The luminal surface of arteries and veins is lined with endothelium, a single layer of cells that maintains a strong yet selectively permeable barrier between the blood and the surrounding tissue. In homeostasis, the endothelium prevents leakage of plasma and other blood components by forming interconnected cell–cell junctions.^1,2^ A key component of these structures is the adherens junction, which is formed by the transmembrane receptor vascular endothelial (VE)-cadherin, a mediator of homotypic adhesion between adjacent cells.^3^ During inflammation, the endothelial cells capture circulating leukocytes and direct them to exit the bloodstream to reach the affected tissue.^4–6^ The transmigration process is controlled by a sequence of receptor–ligand interactions between the leukocytes and endothelium, including heterotypic adhesions mediated through selectins, ICAM-1, VCAM-1 and integrin receptors.^7–9^

VE-cadherin plays a crucial role in inflammation by regulating the integrity of endothelial cell–cell junctions and the paracellular extravasation of leukocytes.^6^ The regulation of VE-cadherin-based junctions during transmigration involves various signal transduction-induced phosphorylation and ubiquitination events at the cytoplasmic domain of VE-cadherin, which modulates its endocytosis and the interaction of the junctional complex with the actin cytoskeleton.^10–12^ VE-cadherin-based junctions are transiently displaced during leukocyte transmigration.^13–15^ In addition, the interaction of the VE-cadherin complex with the actin cytoskeleton is key for junction remodeling during leukocyte extravasation and endothelial barrier recovery afterward.^16,17^

The extracellular domain of VE-cadherin mediates the homotypic binding between adjacent cells. Intriguingly, the human VE-cadherin protein contains two arginine-glycine-aspartate (RGD) motifs that are evolutionary conserved among primates: one in the second and one in the third extracellular calcium-dependent cadherin-like binding domain.^18,19^ RGD motifs, often present in extracellular matrix proteins, are well-known potential integrin-binding sites particularly for the β1, β3, and β5 families.^20–22^ A variety of these RGD-binding integrins are expressed on the surface of endothelial cells, as well as on monocytes.^23^ Leukocytes express integrin α4β1 (VLA-4), which in addition to its function as a receptor for the inflammatory adhesion protein VCAM-1, can bind to RGD-containing ligands.^24,25^ Furthermore, endothelial cells express various β1 integrins, which are required for the proper organization of VE-cadherin-based junctions in the developing vasculature,^26^ highlighting that there is close crosstalk between these adhesion receptors. Although, at present there is no direct evidence for a molecular interaction between VE-cadherin and integrin proteins, the aforementioned observations suggest that the RGD motifs of VE-cadherin may serve as ligands for integrins on endothelial and/or leukocytes.

Here, we investigated the potential functional relevance of the RGD motifs in VE-cadherin. Endothelial cells in which endogenous VE-cadherin was replaced with variants in which the RGD motifs were mutated to nonfunctional RGE sequences, still formed normal adherens junctions and maintained endothelial barrier function. Furthermore, we show that the RGD motifs of VE-cadherin are not needed for interactions between monocytes and endothelial cells during transmigration. Together, this work provides evidence that VE-cadherin’s RGD motifs are not of major importance to junction-dependent endothelial functions.

## Materials and methods

### Cells

Cord blood outgrowth endothelial cells (BOECs^27,28^), isolated according to protocol,^29^ were cultured at 37°C with 5% CO_2_ in endothelial growth medium (#c -22,211, Promocell) supplemented endothelial growth factor mix (#C-39216, Promocell), 15% heat-inactivated fetal calf serum (#1156036, Gibco), 100 U/mL penicillin (#11548876, Gibco), and 100 μg/mL streptomycin (#15140122, Gibco). Cells were cultured on fibronectin (FN; source Sanquin) coated surfaces. To obtain knockdown lines, cells were lentivirally transduced with pLKO.1-shRNA targeting the VE-cadherin 3’UTR (previously validated in Malinova et al.^30^ and were selected with 2.5 ng/mL puromycin (#P8833, Sigma-Aldrich) and compared to shControl (shC002) transduced cells. Next, the knockdown cells were transduced with lentivirus containing GFP-tagged VE-cadherin variants. VE-cadherin-GFP overexpression was selected by fluorescence activated cell sorting (FACS).

### DNA plasmids

The VE-cadherin [D238E] mutation was introduced using site directed mutagenesis on a pEGFP-hVE-cadherin-GFP plasmid using 5’-CCTCCGGGGG**GA****G**TCGGGCACGGCC-3’ and 5’-GGCCGTGCCCGA**C****TC**CCCCCGGAGG-3’ primers. The VE-cadherin[D301E] mutation was introduced using 5’-GCATCTTGCGGGGC**GA****G**TACCAGGACGCTTTCAC-3’ and 5’-GTGAAAGCGTCCTGGTA**C****TC**GCCCCGCAAGATGC-3’ primers. The generated VE-cadherin sequences were next amplified using PCR with primers 5’-TACATCTACGTATTAGTCATCGCTA-3’ and 5’- CCTCTACAAATGTGGTATGGCTGATTATGATC −3’ and inserted into a pLV-CMV-Puro lentiviral plasmid using restriction-ligation protocols with enzymes SnaBI, XbaI, NheI, PstI, and XhoI. Resulting pEGFP and pLV constructs were all verified by Sanger sequencing.

### Western blotting

Cells were lysed in Laemmli reduced sample buffer with 4% ß-mercaptoethanol, denatured for 10 min at 96°C and loaded on 4%–12% gradient SDS-page gels (NW042125BOX, Thermo Fisher Scientific) and run according to the manufacturer’s instructions. After semi-dry transfer onto ethanol-activated PVDF membranes and blocking in 3% bovine serum albumin (BSA; A8806-5 G, Sigma Aldrich) in Tris-buffered saline (TBS; Sigma Aldrich) for 45 min at room temperature (RT), blots were incubated overnight at 4°C in primary antibody in TBS + 3%BSA: rabbit anti-VE-cadherin (D87F2, Cell Signaling Technology), mouse anti-GFP (SC-9996, Santa Cruz), and anti-ß-actin (#4967, Cell Signaling Technology); washed 3 times for 5 min in TBS; incubated at RT in secondary antibody in TBS + 3%BSA: anti-rabbit-HRP (170–6515, Biorad) or anti-mouse-HRP (172–1011, Biorad); washed another 3 times for 5 min in TBS; and imaged using enhanced chemiluminescence detection (34580, Thermo Fisher Scientific) on an Amersham ImageQuant 800 GxP machine (29653452, Cytiva). Protein band intensity was quantified using the FIJI/ImageJ Gel Analyser plugin.

### Electric cell-substrate impedance sensing (ECIS)

Endothelial barrier function was measured using ECIS as previously described.^31^ In short, gold electrodes (8w10E+, #72040, Applied BioPhysics) were treated with 10 mm L-cysteine (Sigma Aldrich) in saline for 15 min at RT and FN-coated for 2 h at 37°C. Impedance measuring at 4,000 hz to assess cell–cell junction integrity and at 16,000 hz to assess cell-extracellular matrix adhesion^32–36^ was performed for 50 h, starting immediately after seeding of 120,000 BOECs per chamber, using the ZTheta machine (Applied BioPhysics). Data analysis was performed in GraphPad Prism.

### Monocyte isolation

Pan-monocytes were isolated from whole-peripheral blood from healthy voluntary donors that signed an informed consent from the Amsterdam University Medical Center medical ethical committee in accordance with the rules and regulations within the Netherlands, based on the declaration of Helsinki and the guidelines for good clinical practice. Within 1 h after donation, blood was diluted (1:1) in RT phosphate buffered saline (PBS; #M09001/02, Fresenius Kabi) with 1:10 TNC, transferred onto 1.076 g/mL Percoll separation medium at RT, and separated by centrifuging at RT for 20 min at 800 G with start and brake at setting 3. After Percoll separation, the ring fraction was taken, and erythrocytes were lysed in ice-cold buffer (water for injection with 155 mm NH_4_Cl, 10 mm KHCO_3_, 0.1 mm EDTA (all Sigma-Aldrich)) on ice for 25 min. Monocytes were then isolated using the human pan-monocyte isolation kit (#130-096-537, Miltenyi Biotech) according to the manufacturer’s protocol and kept at 37°C for the duration of the experiment.

### Monocyte transmigration under physiological flow

For flow assays, 45,000 BOECs were seeded per channel of FN-coated ibidi µ-slides VI^0.4^ (#80666, ibidi, Munich, Germany) and grown for 48 h until confluence and stimulated for 4 h with 10 ng/mL recombinant human TNFα (300-01A, Peprotech) in endothelial culture medium. During microscopy at 37°C and 5% CO_2_, ibidi slide flow channels were connected to a pump system providing a laminar flow of 0.8 dyne/cm^2^ of 37°C HEPES++ buffer (20 mm HEPES, 132 mm NaCl, 6 mm KCl, 1 mm MgSO_4_, 1.4 mm K_2_HPO_4_ (pH 7.4), 1 mm CaCl_2_, 5 mm D-glucose (all Sigma-Aldrich) and 0.4%w/v human serum albumin (Sequence). Cells received laminar flow for 2 min before 1 million monocytes were introduced into the system. After monocyte introduction, transmigration was captured every 5 s during 15 min on two mid-channel positions using an Axiovert 200 M widefield microscope with a TL Halogen lamp at 6.06 V exposing for 32 ms, detected through a 10× DIC NA0.30 Air objective (Zeiss) by an AxioCam ICc3 camera (Zeiss). In addition, immediately after the 15 min, an image overview of the channels was generated by stitching a tile scan of 4 × 6 frames. These images were used to quantify total adhesion and transmigration of monocytes. Tested endothelial monolayers were randomized to exclude variations of leukocyte freshness post-isolation. Analysis was performed as previously described in Grönloh et al.^37^

### Immunofluorescence

For immunofluorescence stainings, 90,000 BOECs were grown until 48 h at confluence on FN-coated ibidi µ-slides VI^0.4^ (#80666, ibidi, Munich, Germany) and fixed in 4% PFA in PBS with 1 μg/mL CaCl_2_ and 0.5 μg/mL MgCl_2_ (PBS++) for 5 min at 37°C. Fixed cells were permeabilized using 0.01% Triton (X100, Sigma Aldrich) in PBS++ for 10 min at RT, blocked in 3% BSA in PBS++ at RT for 45 min, stained with primary antibody for 1 h at RT and secondary antibodies for 45 min at RT. Alexa Fluor 488-conjugated VE-cadherin mouse monoclonal antibody was purchased from ABCAM (ab272345; diluted 1:250). Anti-α-catenin mouse monoclonal antibody was purchased from Enzo Life Sciences (ALX-804-101; diluted 1:300). Alexa Fluor 594-conjugated chicken anti-mouse antibody was purchased from Invitrogen (A21201; diluted 1:400). Hoechst 33342 was purchased from Molecular Probes (H-1399; diluted 1:25,000). PromoFluor 415-Phalloidin was purchased from PromoKine (PK-PF415-7-01, diluted 1:200). Fluorescence was captured on a Nikon Eclipse TI microscope with SOLA SEII light source, 60 × 1.49NA Apo TIRF oil objective, standard NIKON filter cubes, using a Andor Zyla 4.2 plus sCMOS camera. Images were enhanced for display using an unsharp mask filter and adjusted for brightness and contrast using FIJI/ImageJ software.

### Statistical analysis

GraphPad Prism was used for the statistical analysis of all data. For ECIS data, all data points were corrected for background signal of medium only conditions and technical duplicates were averaged per biological replicate. Western blots were corrected for background, for total protein loading, and related to control band intensity within a biological replicate condition. Comparison between multiple groups was made using one-way ANOVA in combination with Dunnett’s post-hoc test for multiple comparisons and a D’Agostino-Pearson test for normality. All graphs show mean and standard deviation and p-values are indicated in the graphs.

## Results

### Replacing endogenous VE-cadherin with VE-cadherin-[RGD>RGE] variants in endothelial cells

**Figure 1.**
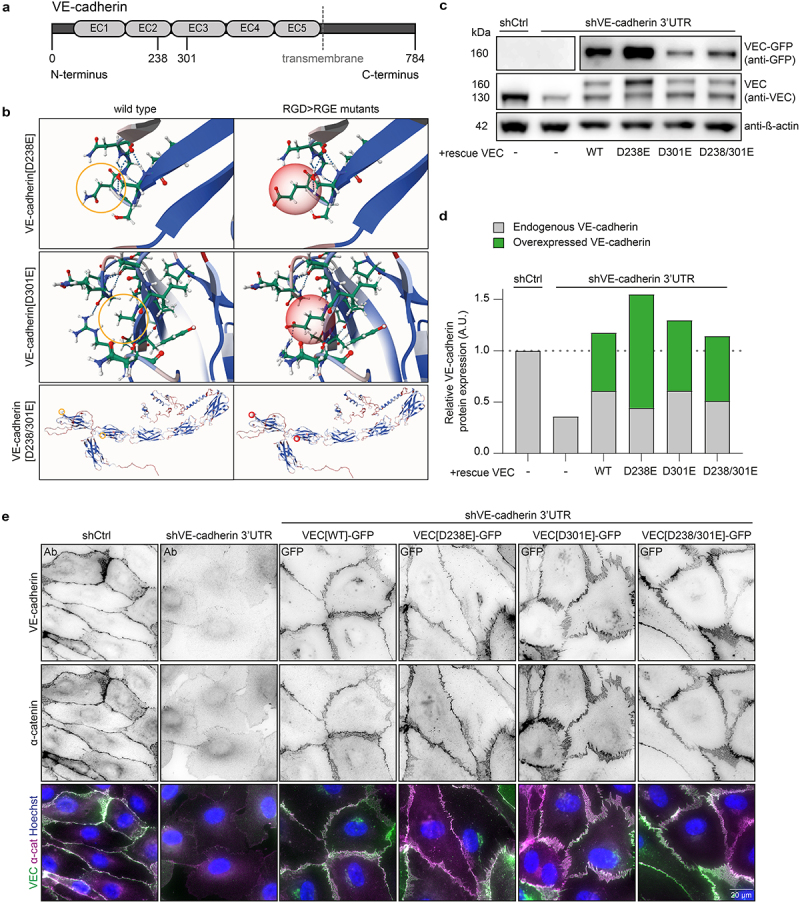
Replacing endogenous VE-cadherin with VE-cadherin-[RGD>RGE] variants in endothelial cells. (a) Human VE-cadherin contains two RGD sites in its extracellular EC2 and EC3 domains. The locations of the D>E mutations in the RGD domains are at amino acid positions 238 and 301. (b) 3D visualization of the amino acid substitution using the MutationExplorer web tool.^38^ (c) Representative Western blot analysis of lysates derived from wild-type BOECs and cells transduced with control shRNA or shVE-cadherin-3’UTR and subsequent rescue lines. Blots are probed for VE-cadherin, GFP and ß-actin as loading control. See Supplemental Figure S2 for corresponding full scans of the blots. (d) Analysis of VE-cadherin blot from (C). Graph shows VE-cadherin expression levels, signal corrected for background and relative to expression of endogenous VE-cadherin in control shRNA-treated cells. (e) Representative immunofluorescence images of BOECs transduced shVE-cadherin-3’UTR to deplete endogenous VE-cadherin and rescued with the indicated VE-cadherin-GFP variants (GFP, green). shCtrl and shVE-cadherin cells were stained for endogenous VE-cadherin (Ab, green). All cells were stained for α-catenin (magenta) and Hoechst (blue).

Mammalian VE-cadherin contains two RGD motifs that are located on the extracellular part of the protein within the second and third cadherin domain (Figure 1(a)). RGD motifs, as adhesion ligand, can be rendered inactive by substituting the aspartic acid for glutamic acid (D>E), which retains the motif’s negative charge while altering its steric composition^39^ (Figure 1(b)). We generated lentiviral plasmids expressing GFP-tagged human wild-type VE-cadherin (WT), its variants in which the individual RGD motifs are converted to nonfunctional RGE sequences (D238E and D301E), and a double mutated variant in which both RGD motifs were converted to RGE (D238/301E). These point mutations would prevent potential integrin interactions with the RGD motifs while keeping the rest of the extracellular VE-cadherin protein structure intact. To assess the function of each VE-cadherin RGD>RGE variant in endothelial cells (ECs), we first knocked down the expression of endogenous VE-cadherin by using lentiviral shRNAs targeting the 3′-untranslated region (3′-UTR) of the CDH5 messenger RNA in cord blood outgrowth endothelial cells (BOECs) (Figure 1(c,d)). VE-cadherin endogenous protein depletion was confirmed by Western blot (Figure 1(c,d)) and resulted in a loss of adherens junctions as verified by immunofluorescent (IF) imaging of VE-cadherin and α-catenin (Figure 1(e)). Next, the VE-cadherin knockdown ECs were rescued by stable expression of lentivirally transduced wild-type (WT) VE-cadherin-GFP, or the RGE variants, and GFP-based fluorescence-activated cell sorting (Figure 1(c,d)). Total VE-cadherin protein levels after rescue were comparable to endogenous levels (Figure 1(c, d); Supplemental Figure S1A, Supplemental Figure S2). IF imaging of the generated endothelial cell lines showed that the VE-cadherin [D238E], VE-cadherin [D301E] and VE-cadherin [D238/301E] protein variants all efficiently restored the formation of adherens junctions, to similar extent as VE-cadherin [WT] (Figure 1(e)). In addition, no obvious differences were detected in junctional localization of the VE-cadherin variants or the actin cytoskeletal organization of the generated cell lines (Supplemental Figure S1B), nor in the localization of the core adherens junction protein α-catenin (Figure 1(e)).

### The VE-cadherin RGD motifs are dispensable for endothelial barrier function

**Figure 2.**
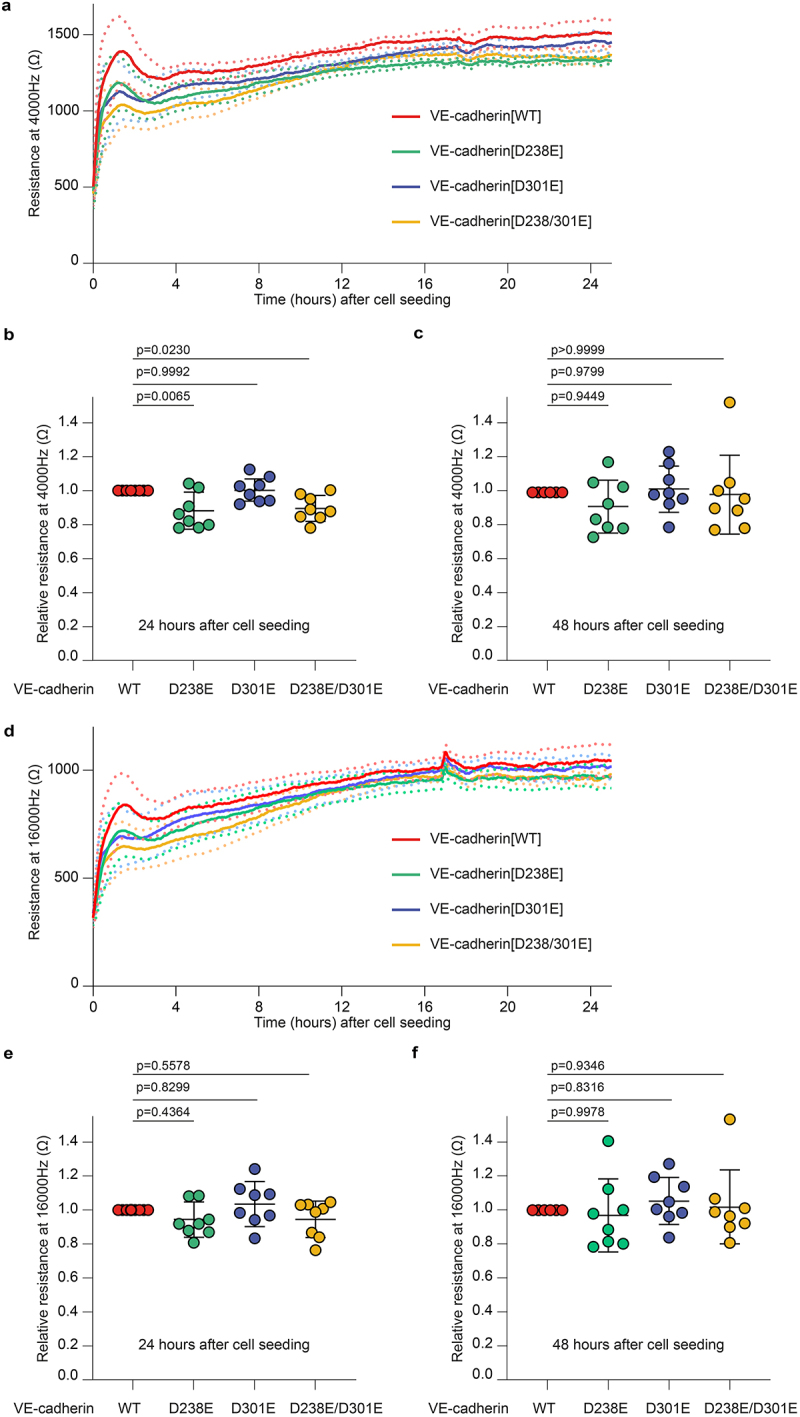
VE-cadherin RGD motifs are dispensable for endothelial barrier function. (a) Line graph showing the average resistance (±SEM, dotted lines) measured with ECIS at 4,000 hz of indicated BOEC monolayers over time. Data from *n* = 8 independent experiments. (b,c) Graphs showing the relative, compared to VE-cadherin[WT], resistance at 4,000 hz 24 (b) and 48 (c) hours after cell seeding. Data from *n* = 8 independent experiments, mean and SD shown, ANOVA with Dunnett’s multiple comparison test, p-values indicated. (d) Line graph showing the average resistance (±SEM, dotted lines) measured with ECIS at 16,000 hz of BOEC monolayers over time. Data from *n* = 8 independent experiments. (e,f) Graphs showing the relative, compared to VE-cadherin[WT], resistance at 16,000 hz 24 (e) and 48 (f) hours after cell seeding data from *n* = 8 independent experiments, mean and SD shown, ANOVA with Dunnett’s multiple comparison test, p-values indicated.

**Figure 3.**
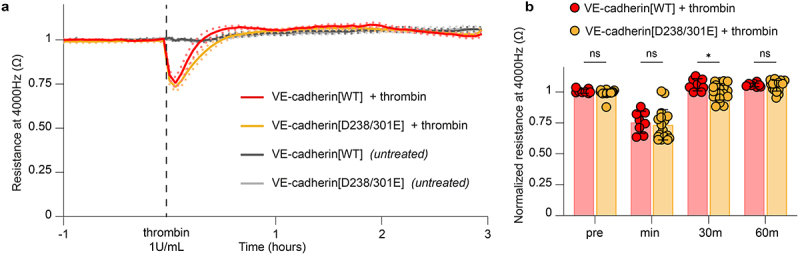
Normal thrombin-induced permeability response in VE-cadherin [D238/301E] cells. (a) Line graph showing the average resistance (±SEM, dotted lines) measured with ECIS at 4,000 hz of indicated BOEC monolayers over time following treatment with the permeability factor human plasma-derived thrombin (used at 1 U/mL). Data are normalized to the baseline values prior to thrombin treatment and are derived from *n* = 4 independent experiments. (b) Graphs showing the relative, compared to VE-cadherin[WT], resistance at 4,000 hz at baseline before thrombin stimulation (*pre*), at the lowest barrier level after thrombin treatment (*min*), and 30 and 60 minutes after thrombin treatment. Data from *n* = 4 independent experiments performed in duplo, mean and SD shown, ANOVA with Dunnett’s multiple comparison test, *p < 0.05.

VE-cadherin mediates homotypic binding in trans between adjacent endothelial cells through the first extracellular cadherin (EC) domain.^40^ The RGD motifs are located within the second and third EC domains, potentially affecting the adhesive function of VE-cadherin. In addition, we hypothesized that the RGD motifs may contribute to barrier function through heterotypic interactions between endothelial cells binding to endothelial ß1 and ß3 integrins. To examine barrier function in the RGD>RGE cell lines we performed electric cell-substrate impedance sensing (ECIS) measurements at different frequencies: 4,000 hz to assess the tightness of cell–cell junctions in the respective endothelial monolayers, and 16,000 hz to assess cell adhesion to the substrate.^31,34,35^ These experiments showed that the VE-cadherin-depleted cells show severely impaired barrier function as expected (Supplemental Figure S1C-E), while cell lines rescued with VE-cadherin WT or RGD>RGE variants form stable barriers (Figure 2(a)). Cells expressing VE-cadherin RGD>RGE, in particular VE-cadherin [D238/301E], display a slight delay in the initial phase of endothelial barrier formation when compared to VE-cadherin [WT] (Figure 2(a)). At 24 h after seeding, all cell lines reached maximal barrier function, indicating the establishment of confluent monolayers (Figure 2(b)). We observed that monolayers formed by VE-cadherin [D238E] and VE-cadherin [D238/D301E] were slightly less resistant compared to the control. However, no significant differences in the barrier function of the endothelial monolayers were detected at 48 h post-cell seeding (Figure 2(c)). Endothelial cells expressing VE-cadherin RGD>RGE displayed only a minor delay in initial cell-substrate adhesion within 2–4 h post-seeding, based on impedance sensing at a frequency of 16,000 hz (Figure 2(d)), but no significant differences were measured at 24 and 48 h after seeding (Figure 2(e,f)). To further test the role of the RGD motifs in adherens junction dynamics, we next challenged endothelial monolayers with the permeability factor thrombin.^31,41^ These experiments showed that VE-cadherin [D238/301E] expressing monolayers responded normally to thrombin stimulation, as visualized by the temporary loss of barrier function (Figure 3(a)). There was a minor delay in the barrier recovery phase at 30 min after thrombin treatment (Figure 3(b)). Hence, we conclude that the extracellular RGD motifs might minimally support VE-cadherin-based junction formation, but do not contribute substantially to cell–cell junction stability over time, nor are they critical for adhesion of endothelial cells to the basal substrate.

### VE-cadherin’s RGD motifs do not regulate monocyte adhesion and transendothelial migration

**Figure 4.**
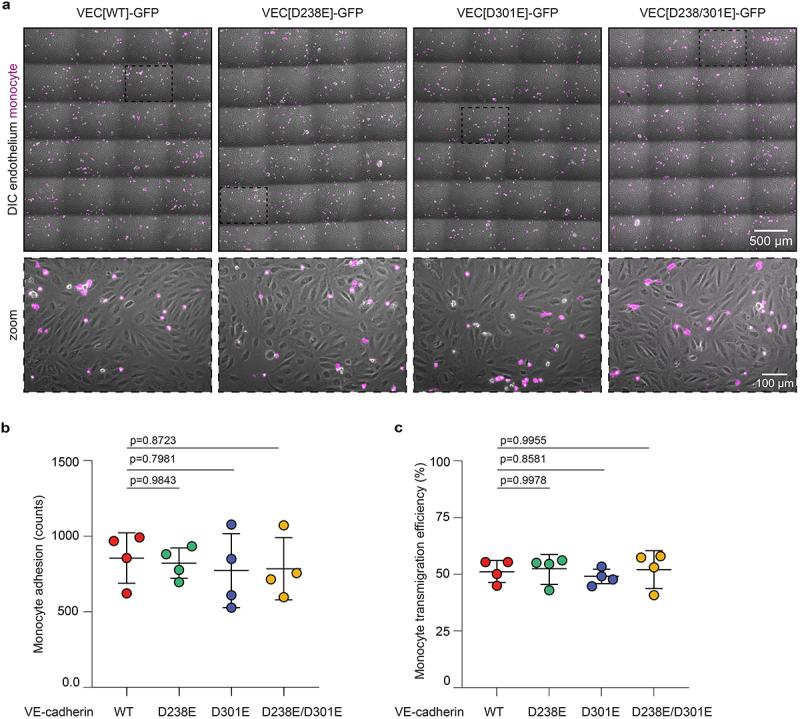
VE-cadherin’s RGD motifs do not regulate monocyte adhesion and transendothelial migration. (a) Representative images (4×6 stitched together) taken after 10 minutes of flowing monocytes (magenta) over TNFα-stimulated inflamed wild type and RGD>RGE mutant VE-cadherin-expressing BOEC monolayers (gray) under physiological flow. (b) Graph indicating absolute monocyte adhesion to wild type and RGD>RGE mutant VE-cadherin-expressing BOEC monolayers under physiological flow. Data from *n* = 4 independent experiments, mean and SD shown, ANOVA with Dunnett’s multiple comparison test, p-values indicated. (c) Graph showing monocyte transendothelial migration efficiency as the percentage of all adhered monocytes. Data from *n* = 4 independent experiments, ANOVA with Dunnett’s multiple comparison test, p-values indicated.

As the RGD motif is a recognition site for various integrins that are expressed by monocytes,^23^ we next investigated whether VE-cadherin might control the adhesion and/or transendothelial migration of these leukocytes via its RGD motifs. To assess this, the VE-cadherin variant endothelial monolayers were grown in fibronectin-coated channel slides and stimulated with inflammatory mediator tumor necrosis factor (TNF)α. Next, monocytes isolated from healthy donor blood were allowed to transmigrate across the inflamed monolayers under physiological flow of 0.8 dyne/cm^2^ (Figure 4(a)). These experiments indicate that endothelial cells expressing VE-cadherin RGD>RGE-mutants retained their ability to facilitate capture and rolling of monocytes (Figure 4(a,b)). Moreover, monocytes transmigrated with equal efficiency across the different endothelial monolayers (Figure 4(c)). Together, these data imply no major role for the RGD motifs of VE-cadherin in monocyte recruitment or transmigration.

## Discussion

The presence of the RGD motifs in the extracellular domain of VE-cadherin is a seemingly overlooked molecular property with a possible role in endothelial cells. In this study, we replaced the endogenous VE-cadherin protein with RGD>RGE nonfunctional variants to investigate this aspect. Our results show that the VE-cadherin RGD motifs are not crucial for key endothelial functions such as adherens junction formation, maintenance of endothelial barrier function and monocyte transendothelial migration.

The presence of the RGD motif in various extracellular ligands promotes cell adhesion, migration, and signaling in a multitude of cell types, including endothelial cells and leukocytes, monocytes in particular.^20,22,39,42^ The RGD motif, often part of extracellular matrix components such as fibronectin, vitronectin and fibrinogen, mediates adhesion by ß1 and ß3 integrins. Other integrin subtypes are well known to control the rolling, adhesion and transendothelial migration cascade of leukocytes. These steps rely for instance on the function of leukocyte-expressed LFA-1 and Mac-1, which both contain the ß2 integrin subunit, but do not interact to ligands through RGD. Our data show that the RGD motifs of VE-cadherin do not significantly alter the interaction between monocytes and endothelial cells. This indicates that RGD-binding integrins on monocytes, such as αvβ3 or α5β1, do not use VE-cadherin’s RGD motifs during transmigration. An explanation for why these RGD motifs do not affect monocyte adhesion might be the potential steric inaccessibility of the peptide sequence within the VE-cadherin extracellular domain through posttranslational modifications in the extracellular domain. For instance, decreasing VE-cadherin N-glycosylation has been shown to promote the adhesion of monocytes.^43^ The RGD motifs might also be inaccessible as a result of the tertiary structure of the folded VE-cadherin protein within the adhesion complex, or because of the tight cell–cell contact organization. Adherens junctions are formed by VE-cadherin *trans*-dimerization through the first cadherin domain of proteins on adjacent cells,^44^ as well as *cis-*dimerization between proteins of the same cell via the fourth cadherin domain^40^ resulting in the formation of cadherin clusters.^45^ Cluster density might also shield the RGD motifs from passing leukocytes, hence preventing specific monocyte recruitment.

Another explanation for the dispensable role of VE-cadherin’s RGD motifs is that for optimal adhesion the RGD needs synergistic domains to bind to integrins. In the field of functionalized biomaterials, it has been reported that the isolated RGD motif is less potent to induce integrin-signaling compared to RGD within the context of native proteins.^46^ The cooperation with other protein domains also explains how the RGD peptide can bind different integrins and induce differential signaling.^47^ Examples of such co-motifs are the amino acid sequence PHSRN that allows fibronectin-RGD to specifically activate integrin α5ß1,^48,49^ and the sequence NGR that specifically binds αvß3.^50^ Furthermore, larger protein chains like the 140 amino acid FAS1 domain promote RGD-αvß3 binding.^51^ Altogether, our data suggest that VE-cadherin does not contain the proper protein structure and composition to unlock the adhesive potential of its extracellular RGD sites.

In summary, by using mutagenesis to disrupt the RGD motifs, combined with assays that examined cell–cell junctions, cell adhesion, endothelial barrier function and monocyte transmigration, our results suggest that there is no major role for VE-cadherin’s RGD motifs in in vitro endothelial cells. To fully rule out a possible function of VE-cadherin’s RGD motifs in the vasculature one could generate transgenic zebrafish or mouse models. Using such transgenic models one can determine whether the RGD motifs of VE-cadherin are needed for vascular development or vascular maintenance within a physiologically perfused organism. However, with the obtained insights from the in vitro cells generated in this study, it currently lacks sufficient rationale to make that effort.

## Supplementary Material

Supplemental Material

## References

[1] Bazzoni G, Dejana E. Endothelial cell-to-cell junctions: molecular organization and role in vascular homeostasis. Physiol Rev. 2004;84:1–12. doi:10.1152/physrev.00035.2003. 15269339

[2] Hartsock A, Nelson WJ. Adherens and tight junctions: structure, function and connections to the actin cytoskeleton. Biochim et Biophys Acta (BBA) - Biomembr. 2008;1778:660–669. doi:10.1016/j.bbamem.2007.07.012. PMC2682436 17854762

[3] Dejana E, Orsenigo F, Lampugnani MG. The role of adherens junctions and ve-cadherin in the control of vascular permeability. J Cell Sci. 2008;121:2115–2122. doi:10.1242/jcs.017897. 18565824

[4] Ley K, Laudanna C, Cybulsky MI, Nourshargh S. Getting to the site of inflammation: the leukocyte adhesion cascade updated. Nat Rev Immunol. 2007;7:678–689. doi:10.1038/nri2156. 17717539

[5] Nourshargh S, Alon R. Leukocyte migration into inflamed tissues. Immunity. 2014;41:694–707. doi:10.1016/j.immuni.2014.10.008. 25517612

[6] Vestweber D. How leukocytes cross the vascular endothelium. Nat Rev Immunol. 2015;15:692–704. doi:10.1038/nri3908. 26471775

[7] Aman J, Margadant C. Integrin-dependent cell–matrix adhesion in endothelial health and disease. Circ Res. 2023;132:355–378. doi:10.1161/CIRCRESAHA.122.322332. 36730379 PMC9891302

[8] Dejana E, Raiteri M, Resnati M, Lampugnani MG. Endothelial integrins and their role in maintaining the integrity of the vessel wall. Kidney Int. 1993;43:61–65. doi:10.1038/ki.1993.11. 8433570

[9] Mitroulis I, Alexaki VI, Kourtzelis I, Ziogas A, Hajishengallis G, Chavakis T. Leukocyte integrins: role in leukocyte recruitment and as therapeutic targets in inflammatory disease. Pharmacol Ther. 2015;147:123–135. doi:10.1016/j.pharmthera.2014.11.008. 25448040 PMC4324083

[10] Arif N, Zinnhardt M, Nyamay’antu A, Teber D, Brückner R, Schaefer K, Li Y, Trappmann B, Grashoff C, Vestweber D. PECAM‐1 supports leukocyte diapedesis by tension‐dependent dephosphorylation of ve‐cadherin. EMBO J. 2021;40:e106113. doi:10.15252/embj.2020106113. 33604918 PMC8090850

[11] Wessel F, Winderlich M, Holm M, Frye M, Rivera-Galdos R, Vockel M, Linnepe R, Ipe U, Stadtmann A, Zarbock A, et al. Leukocyte extravasation and vascular permeability are each controlled in vivo by different tyrosine residues of ve-cadherin. Nat Immunol. 2014;15:223–230. doi:10.1038/ni.2824. 24487320

[12] Wilkens M, Holtermann L, Stahl A-K, Stegmeyer RI, Nottebaum AF, Vestweber D. Ubiquitination of ve-cadherin regulates inflammation-induced vascular permeability in vivo. EMBO Rep. 2024;25:4013–4032. doi:10.1038/s44319-024-00221-7. 39112792 PMC11387630

[13] Arts JJ, Mahlandt EK, Grönloh ML, Schimmel L, Noordstra I, Gordon E, Van Steen AC, Tol S, Walzog B, Van Rijssel J, et al. Endothelial junctional membrane protrusions serve as hotspots for neutrophil transmigration. eLife. 2021;10:e66074. doi:10.7554/eLife.66074. 34431475 PMC8437435

[14] Liu Y, Shaw SK, Ma S, Yang L, Luscinskas FW, Parkos CA. Regulation of leukocyte transmigration: cell surface interactions and signaling events. J Immunol. 2004;172:7–13. doi:10.4049/jimmunol.172.1.7. 14688302

[15] Van Buul JD, Voermans C, Van Den Berg V, Anthony EC, Mul FPJ, Van Wetering S, Van Der Schoot CE, Hordijk PL. Migration of human hematopoietic progenitor cells across bone marrow endothelium is regulated by vascular endothelial cadherin. J Immunol. 2002;168:588–596. doi:10.4049/jimmunol.168.2.588. 11777950

[16] Martinelli R, Kamei M, Sage PT, Massol R, Varghese L, Sciuto T, Toporsian M, Dvorak AM, Kirchhausen T, Springer TA, et al. Release of cellular tension signals self-restorative ventral lamellipodia to heal barrier micro-wounds. J Cell Biol. 2013;201:449–465. doi:10.1083/jcb.201209077. 23629967 PMC3639391

[17] Schulte D, Küppers V, Dartsch N, Broermann A, Li H, Zarbock A, Kamenyeva O, Kiefer F, Khandoga A, Massberg S, et al. Stabilizing the ve-cadherin-catenin complex blocks leukocyte extravasation and vascular permeability: necessity of paracellular diapedesis in vivo. EMBO J. 2011;30:4157–4170. doi:10.1038/emboj.2011.304. 21857650 PMC3199392

[18] Bartolomé RA, Torres S, De Val SI, Escudero-Paniagua B, Calviño E, Teixidó J, Casal JI. Ve-cadherin RGD motifs promote metastasis and constitute a potential therapeutic target in melanoma and breast cancers. Oncotarget. 2017;8:215–227. doi:10.18632/oncotarget.13832. 27966446 PMC5352113

[19] Casal JI, Bartolomé RA. Beyond N-Cadherin, relevance of cadherins 5, 6 and 17 in cancer progression and metastasis. IJMS. 2019;20:3373. doi:10.3390/ijms20133373. 31324051 PMC6651558

[20] Benelli R, Mortarini R, Anichini A, Giunciuglio D, Noonan DM, Montalti S, Tacchetti C, Albini A. Monocyte-derived dendritic cells and monocytes migrate to HIV-Tat RGD and basic peptides. AIDS. 1998;12:261–268. doi:10.1097/00002030-199803000-00003. 9517988

[21] Pytela R, Pierschbacher MD, Argraves S, Suzuki S, Ruoslahti E. [27] arginine-glycine-aspartic acid adhesion receptors, in: methods in Enzymology. Elsevier; 1987. p. 475–489. doi:10.1016/0076-6879(87)44196-7. 2442581

[22] Ruoslahti E, Pierschbacher MD. New perspectives in cell adhesion: RGD and Integrins. Science. 1987;238:491–497. doi:10.1126/science.2821619. 2821619

[23] Languino LR, Ruoslahti E. An alternative form of the integrin beta 1 subunit with a variant cytoplasmic domain. J Biol Chem. 1992;267:7116–7120. doi:10.1016/S0021-9258(19)50545-2. 1551917

[24] Amschler K, Kossmann E, Erpenbeck L, Kruss S, Schill T, Schön M, Möckel SMC, Spatz JP, Schön MP. Nanoscale tuning of VCAM-1 determines VLA-4–dependent melanoma cell plasticity on RGD motifs. Mol Cancer Res. 2018;16:528–542. doi:10.1158/1541-7786.MCR-17-0272. 29222169 PMC5837006

[25] Yusuf-Makagiansar H, Anderson ME, Yakovleva TV, Murray JS, Siahaan TJ. Inhibition of LFA-1/ICAM-1 and VLA-4/VCAM-1 as a therapeutic approach to inflammation and autoimmune disease. Med Res Rev. 2002;22(2):146–167. doi:10.1002/med.10001. 11857637

[26] Yamamoto H, Ehling M, Kato K, Kanai K, Van Lessen M, Frye M, Zeuschner D, Nakayama M, Vestweber D, Adams RH. Integrin β1 controls ve-cadherin localization and blood vessel stability. Nat Commun. 2015;6:6429. doi:10.1038/ncomms7429. 25752958

[27] Ingram DA, Mead LE, Tanaka H, Meade V, Fenoglio A, Mortell K, Pollok K, Ferkowicz MJ, Gilley D, Yoder MC. Identification of a novel hierarchy of endothelial progenitor cells using human peripheral and umbilical cord blood. Blood. 2004;104:2752–2760. doi:10.1182/blood-2004-04-1396. 15226175

[28] Lin Y, Banno K, Gil C-H, Myslinski J, Hato T, Shelley WC, Gao H, Xuei X, Liu Y, Basile DP, et al. Origin, prospective identification, and function of circulating endothelial colony-forming cells in mice and humans. JCI Insight. 2023;8:e164781. doi:10.1172/jci.insight.164781. 36692963 PMC10077473

[29] Martin-Ramirez J, Hofman M, Van Den Biggelaar M, Hebbel RP, Voorberg J. Establishment of outgrowth endothelial cells from peripheral blood. Nat Protoc. 2012;7:1709–1715. doi:10.1038/nprot.2012.093. 22918388

[30] Malinova TS, Angulo-Urarte A, Nüchel J, Tauber M, van der Stoel MM, Janssen V, de Haan A, Groenen AG, Tebbens M, Graupera M, et al. A junctional PACSIN2/EHD4/MICAL-L1 complex coordinates ve-cadherin trafficking for endothelial migration and angiogenesis. Nat Commun. 2021;12(1):2610. doi:10.1038/s41467-021-22873-y. 33972531 PMC8110786

[31] Van Der Stoel MM, Kotini MP, Schoon RM, Affolter M, Belting H-G, Huveneers S. Vinculin strengthens the endothelial barrier during vascular development. Vasc Biol. 2022;5(1):e220012. doi:10.1530/VB-22-0012. PMC9986378 36260739

[32] Benson K, Cramer S, Galla H-J. Impedance-based cell monitoring: barrier properties and beyond. Fluids Barriers CNS. 2013;10:5. doi:10.1186/2045-8118-10-5. 23305242 PMC3560213

[33] Luong JHT, Xiao C, Lachance B, Leabu ŠM, Li X, Uniyal S, Chan BMC. Extended applications of electric cell-substrate impedance sensing for assessment of the structure–function of α2β1 integrin. Analytica (Rome) Acta. 2004;501:61–69. doi:10.1016/j.aca.2003.09.016.

[34] Robilliard LD, Kho DT, Johnson RH, Anchan A, O’Carroll SJ, Graham ES. The importance of multifrequency impedance sensing of endothelial barrier formation using ECIS technology for the generation of a strong and durable paracellular barrier. Biosensors. 2018;8:64. doi:10.3390/bios8030064. 29973526 PMC6163417

[35] Tiruppathi C, Malik AB, Vecchio PJD, GIAEVERt I, Giaever I. Electrical method for detection of endothelial cell shape change in real time: assessment of endothelial barrier function. Proc Natl Acad Sci USA. 1992;89(17):7919–7923. doi:10.1073/pnas.89.17.7919. 1518814 PMC49826

[36] Zhang Z, Huang X, Liu K, Lan T, Wang Z, Zhu Z. Recent advances in electrical impedance sensing technology for single-cell analysis. Biosensors. 2021;11:470. doi:10.3390/bios11110470. 34821686 PMC8615761

[37] Grönloh MLB, Arts JJG, Mahlandt EK, Nolte MA, Goedhart J, Van Buul JD. Primary adhered neutrophils increase JNK1-MARCKSL1-mediated filopodia to promote secondary neutrophil transmigration. iScience. 2023;26:107406. doi:10.1016/j.isci.2023.107406. 37559902 PMC10407253

[38] Philipp M, Moth CW, Ristic N, Tiemann JKS, Seufert F, Panfilova A, Meiler J, Hildebrand PW, Stein A, Wiegreffe D, et al. MutationExplorer: a webserver for mutation of proteins and 3D visualization of energetic impacts. Nucleic Acids Res. 2024;52:W132–W139. doi:10.1093/nar/gkae301. 38647044 PMC11223880

[39] Pierschbacher MD, Ruoslahti E. Cell attachment activity of fibronectin can be duplicated by small synthetic fragments of the molecule. Nature. 1984;309:30–33. doi:10.1038/309030a0. 6325925

[40] Brasch J, Harrison OJ, Ahlsen G, Carnally SM, Shapiro B, Honig L, Shapiro L. Structure and binding mechanism of vascular endothelial cadherin, a divergent classical cadherin. J Mol Biol. 2011;408(1):57–73. doi:10.1016/j.jmb.2011.01.031. 21269602 PMC3084036

[41] Rabiet M-J, Plantier J-L, Rival Y, Genoux Y, Lampugnani M-G, Dejana E. Thrombin-induced Increase in endothelial permeability is associated with changes in cell-to-cell junction organization. ATVB. 1996;16:488–496. doi:10.1161/01.ATV.16.3.488. 8630677

[42] Kamoshida G, Matsuda A, Sekine W, Mizuno H, Oku T, Itoh S, Irimura T, Tsuji T. Monocyte differentiation induced by co-culture with tumor cells involves rgd-dependent cell adhesion to extracellular matrix. Cancer Lett. 2012;315:145–152. doi:10.1016/j.canlet.2011.09.029. 22104730

[43] Zhang L, Ma L, Li J, Lei J, Chen J, Yu C. Ve-cadherin N-glycosylation modified by N-acetylglucosaminyltransferase V regulates ve-cadherin–β-catenin interaction and monocyte adhesion. Exp Phys. 2021;106(9):1869–1877. doi:10.1113/EP089617. 34117813

[44] Navarro P, Caveda L, Breviario F, Mândoteanu I, Lampugnani M-G, Dejana E. Catenin-dependent and -independent functions of vascular endothelial cadherin. J Biol Chem. 1995;270:30965–30972. doi:10.1074/jbc.270.52.30965. 8537353

[45] Troyanovsky SM. Adherens junction: the ensemble of specialized cadherin clusters. Trends In Cell Biol. 2023;33:374–387. doi:10.1016/j.tcb.2022.08.007. 36127186 PMC10020127

[46] Hautanen A, Gailit J, Mann DM, Ruoslahti E. Effects of modifications of the RGD sequence and its context on recognition by the fibronectin receptor. J Biol Chem. 1989;264:1437–1442. doi:10.1016/S0021-9258(18)94206-7. 2521482

[47] Mao Y, Schwarzbauer JE. Accessibility to the fibronectin synergy site in a 3D matrix regulates engagement of α 5β 1 versus α vβ 3 integrin receptors. Cell Commun Adhes. 2006;13(5–6):267–277. doi:10.1080/15419060601072215. 17162669

[48] Altroff H, Schlinkert R, Van Der Walle CF, Bernini A, Campbell ID, Werner JM, Mardon HJ. Interdomain tilt angle determines integrin-dependent function of the ninth and tenth FIII domains of human fibronectin. J Biol Chem. 2004;279:55995–56003. doi:10.1074/jbc.M406976200. 15485890 PMC1626575

[49] Aota S, Nomizu M, Yamada KM. The short amino acid sequence pro-his-ser-arg-asn in human fibronectin enhances cell-adhesive function. J Biol Chem. 1994;269:24756–24761. doi:10.1016/S0021-9258(17)31456-4. 7929152

[50] Leiss M, Beckmann K, Girós A, Costell M, Fässler R. The role of integrin binding sites in fibronectin matrix assembly in vivo. Curr Opin In Cell Biol. 2008;20:502–507. doi:10.1016/j.ceb.2008.06.001. 18586094

[51] Son H-N, Nam J-O, Kim S, Kim I-S. Multiple FAS1 domains and the RGD motif of TGFBI act cooperatively to bind αvβ3 integrin, leading to anti-angiogenic and anti-tumor effects. Biochim et Biophys Acta (BBA) - Mol Cell Res. 2013;1833:2378–2388. doi:10.1016/j.bbamcr.2013.06.012. 23792174

