# Supplementary material for: VE-cadherin RGD motifs are dispensable for cell–cell junctions, endothelial barrier function and monocyte extravasation
Source: Tissue Barriers. 2025 Mar 18;13(4):2478349. doi: 10.1080/21688370.2025.2478349 (PMC12667659; doi:10.1080/21688370.2025.2478349)
Supplement: Supplemental Material [file KTIB_A_2478349_SM4016.zip › Supplementary_Figure_legends.docx]

**Supplementary Figure S1. VE-cadherin endogenous knock-down impairs the endothelial barrier. A)** Quantification of the total expression levels of the VE-cadherin-GFP variants normalized to WT VE-cadherin-GFP. Data from n=4 independent Western blot experiments. **B)** BOECs transduced with shVE-cadherin-3’UTR, rescued with indicated VE-cadherin-GFP variants (green), stained for F-actin using phalloidin (magenta). **C)** Line graph showing the average resistance (±SEM, dotted lines) measured with ECIS at 4000Hz of shControl and shVE-cadherin 3’UTR BOEC monolayers over time. **D-E)** Graphs showing the relative resistance of shVE-cadherin 3’ UTR monolayers, compared to shControl, at 4000Hz 24 (B) and 48 (C) hours after cell seeding. Data from n=4 independent experiments, mean and SD shown, Student’s t-test, ***p<0.001, ****p<0.0001.

**Supplementary Figure S2. Full scans of Western blots from Figure 1C.**
