# Supplementary figures and images for: VE-cadherin RGD motifs are dispensable for cell–cell junctions, endothelial barrier function and monocyte extravasation
Source: Tissue Barriers. 2025 Mar 18;13(4):2478349. doi: 10.1080/21688370.2025.2478349 (PMC12667659; doi:10.1080/21688370.2025.2478349)

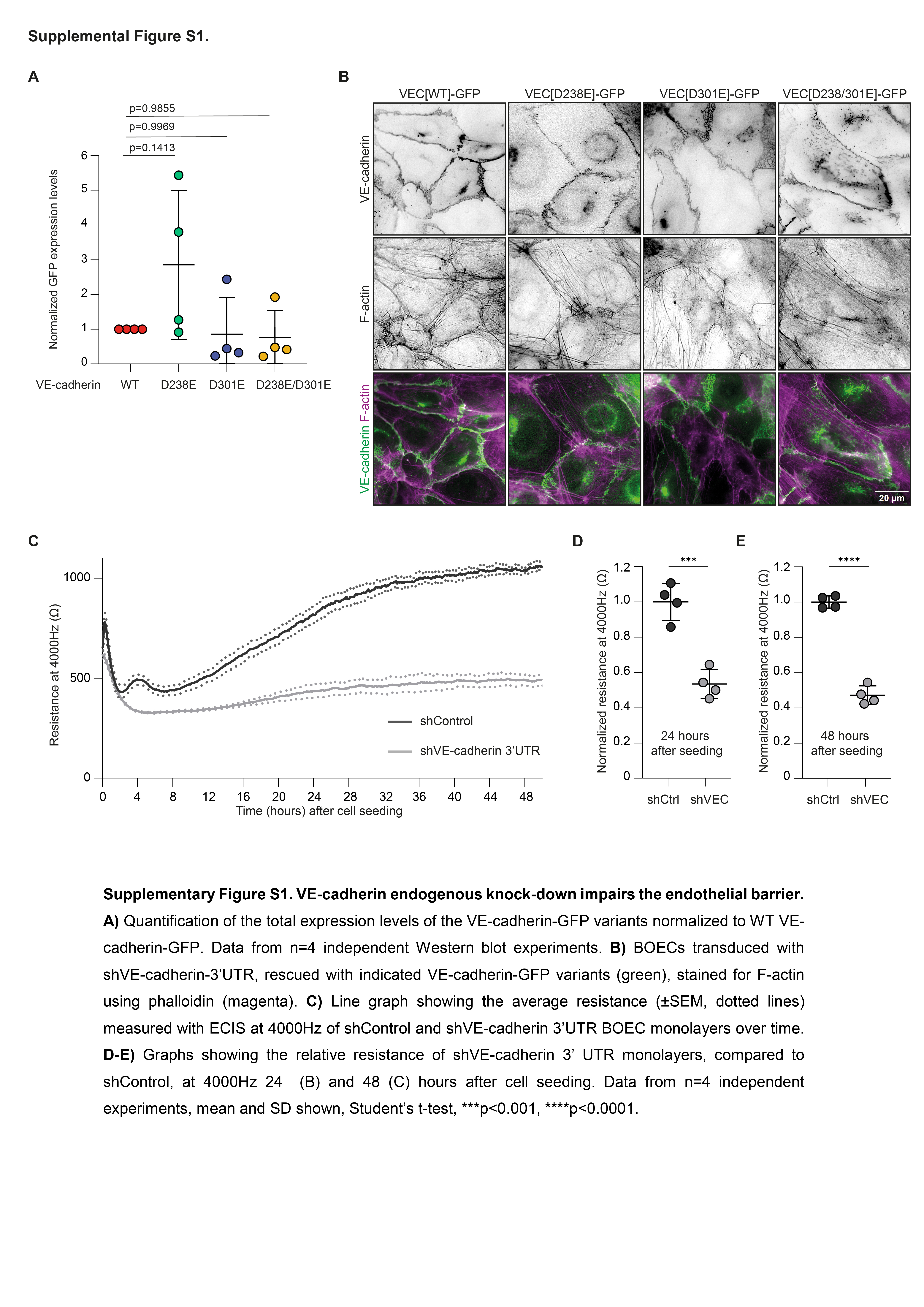

Supplement: Supplemental Material [file KTIB_A_2478349_SM4016.zip › Schoon_et_al_SF1.jpg]

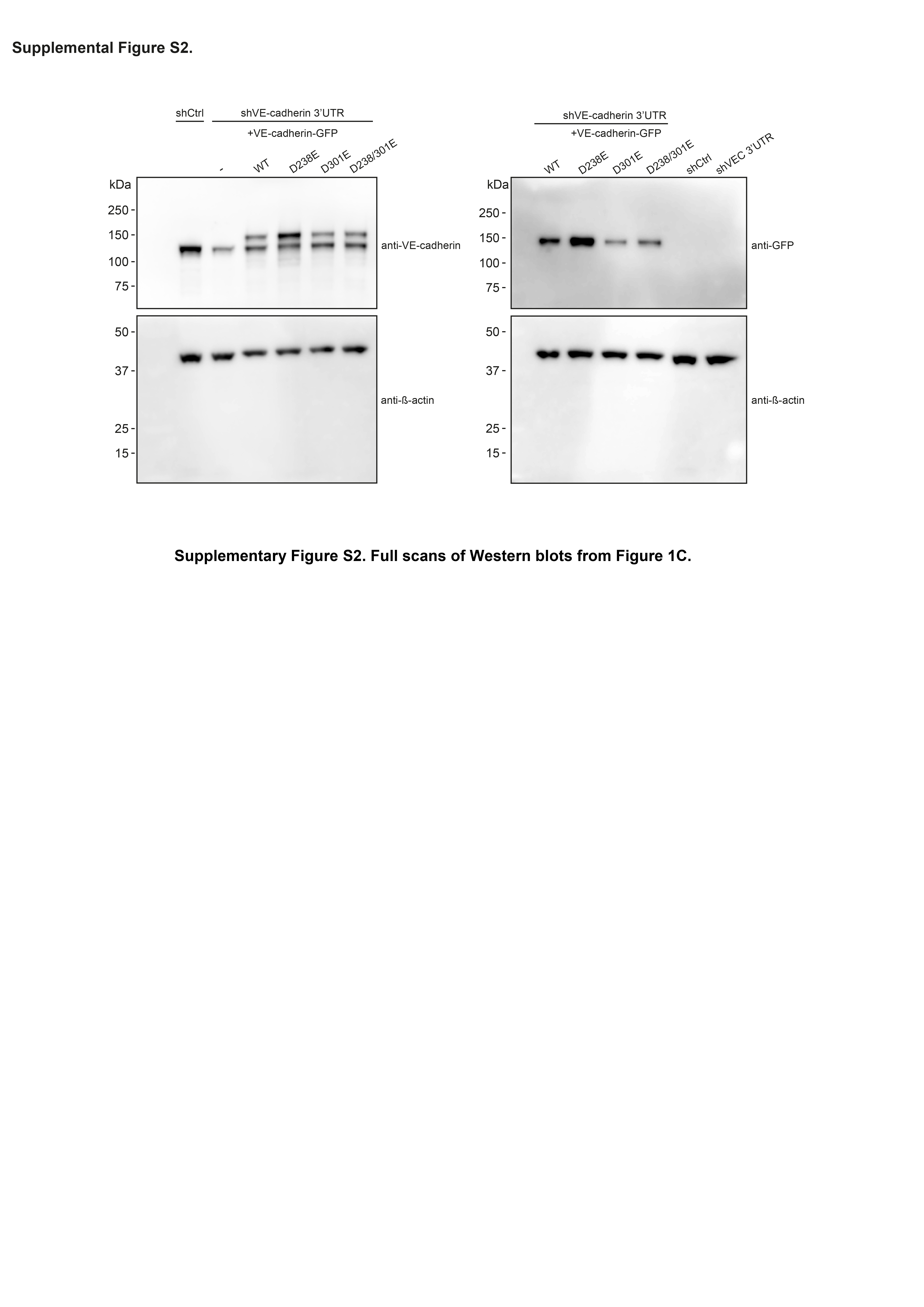

Supplement: Supplemental Material [file KTIB_A_2478349_SM4016.zip › Schoon_et_al_SF2.jpg]
